# Acute Ethanol Intoxication: Αn Overlooked Cause of High Anion Gap Metabolic Acidosis With a Marked Increase in Serum Osmolal Gap

**DOI:** 10.7759/cureus.37292

**Published:** 2023-04-08

**Authors:** Angelos Liontos, Valentini Samanidou, Lazaros Athanasiou, Sebastien Filippas-Ntekouan, Charalambos Milionis

**Affiliations:** 1 1st Department of Internal Medicine, University Hospital of Ioannina, Faculty of Medicine, University of Ioannina, Ioannina, GRC

**Keywords:** serum osmolality, toxic alcohols, high anion gap metabolic acidosis, ethanol intoxication, osmolal gap

## Abstract

Measurement of serum osmolal gap is a useful tool in suspected toxic alcohol ingestion. Normal levels of osmolal gap are typically <10 mOsm/kg). Osmolal gap >20 mOsm/kg is usually caused by ingestion of methanol, ethylene glycol, isopropanol, propylene glycol, diethylene glycol, or organic solvents such as acetone but rarely of ethanol alone. Herein, we describe the case of a severe ethanol intoxication presenting with a marked increase in the osmolal gap. An 18-year-old male was referred to the emergency department of our hospital, in a comatose state, following binge drinking. blood gas analysis revealed a high anion gap metabolic acidosis. In addition, it was found an extremely elevated osmolal gap of 91 mOsm/kg. The increment of the osmolal gap and the high anion gap acidosis could not be attributed to methanol/ethylene glycol intoxication, alcoholic ketoacidosis, or other cause of acidosis. The calculated osmolal concentration of ethanol was 91 mOsm/kg (osmolal concentration of ethanol is equal to the serum ethanol levels (mg/dL) divided by 3.7). Thus, the increase in the osmolal gap was a result of ethanol intoxication solely. Acute, isolated, ethanol intoxication may be a rare cause of a marked increase of osmolal gap with high anion gap metabolic acidosis. Clinicians should be alerted to the possibility of acute ethanol intoxication in a patient presenting with high anion gap metabolic acidosis and an extremely elevated osmolal gap. Toxicologic screen tests should be performed to identify the aetiology of the gap rise and proper therapy should be administered.

## Introduction

Measurement of serum osmolal gap may be a useful tool in everyday clinical practice, especially in patients with suspected ingestion of toxic alcohol. Normal osmolal gap is defined as: measured serum osmolality (sOsm) - calculated sOsm [1]. In everyday clinical practice, the most commonly used formula of calculated sOsm is: sOsm = (2 x Serum {Na (mmol/L)}) + (Glucose {mg/dL})/18 + (Blood urea nitrogen {mg/dL}]/2.8 [2,3].

Typically, osmolal gap levels are <10 mOsm/kg [1]. An osmolal gap >20 mOsm/kg is usually caused by ingestion of low molecular weight water-soluble agents namely: methanol, ethylene glycol, isopropanol, propylene glycol, diethylene glycol or organic solvents such as acetone [1]. On the other hand, an increased osmolal gap >20 mOsm/kg is rarely attributed to the ingestion of ethanol alone [1]. Of note, common causes of metabolic acidosis, ketoacidosis, lactic acidosis, and renal failure usually lead to osmolal gap levels ≤15-20 mOsm/kg [4].

Ingestion of toxic alcohols, may cause organ damage and central nervous system depression; thus, these conditions are treated as medical emergencies [5]. Herein we describe the case of a severe ethanol intoxication presenting with a marked elevation of osmolal gap above expected levels.

## Case presentation

An 18-year-old male was referred to the emergency department of our hospital, in a comatose state, following binge drinking. Patient was reported to consume alcoholic beverages prior to his hospital admission. Exact amount of alcohol ingested could not be verified. Patient’s relatives reported no past medical history or known allergies while the patient was not on any medication on daily basis. The use of illicit drugs was not confirmed either.

Patient’s vitals were documented upon examination; body temperature: 36.7°C, heart rate: 77 beats per minute, blood pressure: 124/75 mm Hg (supine), respiratory rate: 12 breaths per minute with spO2 of 97% on room air (FiO2: 21%). On admission, the patient was responsive only to painful stimuli (9 points on the Glasgow Coma Scale) with equally dilated pupils. No focal neurological deficits were present. Review of the other systems was negative for abnormal findings.

Venus blood gases analysis showed a high anion gap metabolic acidosis (pH=7.29, HCO3=19.9 mmol/L, PCO2=45.6 mmHg, Anion Gap=14 mEq/L). Lactate, urea, creatinine, glucose and creatine kinase levels were within normal limits. Serum ketone levels of beta-hydroxybutyrate (3-OHB) were 0.2 mmol/L. Urine dipstick was positive (+2) for ketones. Laboratory test results are summarized in Table 1.

**Table 1:** Patient's laboratory test results on admission AST: aspartate transaminase, ALT: alanine transaminase, pH: potential of hydrogen, PCO2: partial pressure of carbon dioxide, HCO3: bicarbonate

Variables	Reference Range	On Admission
Hemoglobin (g/dl)	12.0-16.0	16.8
Hematocrit (%)	36.0-46.0	48.5
White-cell count (per mm^3^)	4500-11000	11160
Platelet count (per mm^3^)	150000-450000	253000
Sodium (mmol/L)	135-145	142
Potassium (mmol/L)	3.5-5.3	3.55
Urea (mg/dl)	15-40	23
Creatinine (mg/dl)	0.6-1.2	0.78
Glucose (mg/dl)	70-125	64
Creatine kinase (U/L)	60-400	90
AST (U/L)	10-55	14
ALT (U/L)	10-40	10
Lactic acid (mmol/L)	0.5-2.2	1.9
Phosphorus (mg/dl)	2.6-4.5	4.2
Chloride (mmol/L)	100-108	106
Calcium (mg/dl)	8.5-10.5	8.4
Magnesium (mg/dl)	1.7-2.4	2.04
Venous pH	7.32-7.38	7.29
Venous PCO2 (mmHg)	42-50	45.6
Venous HCO_3_^- ^(mmol/L)	23-27	19.9
Anion Gap (mEq/L)	3-9	14
Ketone level (mmol/L)	<0.6	0.2
Calculated serum Osmolality (mOsm/kg)	285-295	292
Measured serum Osmolality (mOsm/kg)	285-295	383
Osmolal Gap (mOsm/kg)	<10	91

Considering prior binge drinking, alcoholic ketoacidosis was suspected. Thus, next step in the evaluation was the measurement of sOsm. Measured sOsm by freezing point depression, osmometer was 383 mOsm/kg; the calculated sOsm was 292 mOsm/kg, resulting in an osmolal gap of 91 mOsm/kg. Marked increase in osmolal gap raised the suspicion of methanol or ethylene glycol intoxication. On further evaluation, microscopic urinalysis did not show any calcium oxalate crystals. In addition, fundoscopic examination was negative for optic nerve pallor or optic disk edema, bilaterally. Serum and urine toxicology screen tests were obtained.

Intravenous dextrose in water (D/W) 5% solution with multivitamin complex (including thiamine at a dosing of 150 mg/day) and calcium folinate were administrated as per intoxication protocol treatment. Fomepizole, an inhibiting agent of alcohol dehydrogenase which catalyzes the initial steps of ethanol, methanol, and ethylene glycol metabolism to their toxic metabolites, was also administered as per protocol treatment.

Neurological state gradually was restored to normal in the next 48 hours and a concomitant decrease of the osmolal gap to levels <10 mOsm/kg was observed. The patient was discharged 72 hours after admission. Toxicology screen results showed increased serum ethanol levels (336 mg/dL), while methanol and ethylene glycol were not detected.

## Discussion

We report a case of a high anion gap metabolic acidosis with a marked increase in osmolal gap, which raised the suspicion of toxic alcohol ingestion. In our case, the acid-base disorder and increment in osmolal gap were solely attributed to ethanol intoxication.

The most common causes of high anion gap metabolic acidosis with increased osmolal gap include methanol, ethylene glycol, diethylene glycol, propylene glycol or isopropanol intoxication, lactic acidosis, alcoholic or diabetic ketoacidosis, and uremia [4,6,7]. It has been shown that alcoholic ketoacidosis and lactic acidosis may increase the serum osmolal gap by an average of 11 mOsm/kg. However, these levels remain < 20 mOsm/kg [4,8]. Of note, alcoholic ketoacidosis is less common in patients with acute ethanol intoxication, accounting less than 10% of patients [1,9]. Minor to moderate increases in serum osmolal gap are also seen in patients with diabetic ketoacidosis due to the presence of acetone, glycerol, and amino acids. Similar to alcoholic ketoacidosis, osmolal gap levels in these patients remain ≤20 mOsm/kg [1,4,10]. Differential diagnosis of high anion gap metabolic acidosis with increased serum osmolal gap, including causes/factors with their contribution to osmolal gap, are summarized in Table 2 [1,6,11-13].

**Table 2:** Differential diagnosis of high anion gap metabolic acidosis with increased serum osmolal gap N: normal, NA: non-available ↑: elevation, ↓: decline

Cause/Factor	Osmolar Gap	pH	Anion Gap	Ketones	Glucose	Lactate	Contribution to Osmolar Gap
Ethanol only	↑↑	↓	↑	N	N	N	[Ethanol] / 3.7
Methanol	N or ↑	↓	↑	N	N	N	[Methanol] / 3.2
Isopropanol	↑	↓	↑	↑	N	N	[Isopropanol] / 6.0
Ethylene Glycol	↑	↓	↑	N	N	N	[Ethylene glycol] / 6.2
Alcoholic Ketoacidosis	N or ↑ (usually < 20)	↓	↑	↑	N	N	Mainly via ethanol concentration
Diabetic Ketoacidosis	N or ↑ (usually < 20)	↓	↑	↑↑	↑↑	N	[Acetone] / 5.8
Lactic Acidosis	N or ↑ (usually < 20)	↓	↑	N	N	↑↑	NA

Toxic alcohol ingestion increases serum osmolality inversely with its serum concentration and molecular weight. Alcohols with lower molecular weight have the greatest impact on serum osmolality (methanol has the lower molecular weight: 32 g/mol and diethylene glycol the highest: 106 g/mol) [12]. Similarly, ethanol also contributes to serum osmolality and its molar concentration is used to predict the osmolal concentration. In the study by Hoffman et al., it was shown than this concentration can be calculated dividing serum ethanol levels (mg/dL) by 4.6 [14,15]. Rationale for the division by 4.6 is that 1 mg/dL of ethanol equals to 0.22 mmol/L [3]. Similarly, it has been assumed that 1 mmol of ethanol contributes to 1 mOsm/kg of osmolality [3]. However, it has been shown that ethanol contributes more osmoles per kg of water than its molar concentration as it reduces the effective serum water volume [14]. Considering this, in the study by Purssell et al., it was shown that ethanol’s osmolal contribution is more accurately calculated by dividing the serum ethanol concentration (mg/dL) with 3.7 rather than 4.6 [3].

In our case, the osmolar gap was extremely elevated (92 mOsm/kg). Also, lactate, serum creatinine and urea levels were within normal limits. In addition, toxicological screen tests were negative regarding methanol and ethylene glycol concentrations. Thus, the increment of osmolal gap and the high anion gap acidosis could not be attributed to methanol/ethylene glycol intoxication, alcoholic ketoacidosis or other cause of acidosis. Of note, alcoholic ketoacidosis mostly occurs in heavy alcohol abuse following acute decrease in ethanol and food intake [16]. In addition, patient’s serum ketone levels (3-OHB) were low; thus, alcoholic ketoacidosis was excluded. Of note, ethanol is metabolized to acetaldehyde and further to acetic acid in hepatic cells. Acetic acid serves a substrate for ketogenesis through its conversion to acetyl-CoA [17]. Enzymatic pathways lead to the formation of acetoacetate (a ketone body) from acetyl-CoA [16]. Through non-enzymatic decarboxylation or by beta-hydroxybutyrate dehydrogenase, acetoacetate can be transformed into acetone or beta-hydroxybutyrate [16]. However, serum ketone measurement method detects 3-OHB and not acetoacetate [18]. On the other hand, acetoacetate is easily detected in urine with urine ketone stick test [18]. Owing to this, patient’s serum ketone levels were low, although detectable in urine.

In our case, considering the effective molecular weight of ethanol, its contribution to serum osmolality was calculated to be 91 mOsm/kg. Thus, the presence of ethanol found in serum screen tests led to this marked increase in osmolal gap.

Treatment of patients with severe ethanol intoxication (poisoning) requires aggressive supportive measures [19]. Primary objective in this condition is the protection of respiratory airway, as severe intoxication can cause respiratory depression. Intravenous isotonic crystalloid fluids can be administered to patients with symptoms of volume depletion or hypotension [19]. Parenteral thiamine should be administered to all comatose patients due to ethanol intoxication in order to prevent or treat Wernicke's encephalopathy (usual doses in prevention ranging from 100 mg to 250 mg or higher in established encephalopathy) [20].

## Conclusions

Acute, isolated, ethanol intoxication may be a rare cause of a marked increase of osmolal gap with high anion gap metabolic acidosis. Clinicians should be alerted to the possibility of acute ethanol intoxication in a patient presenting with high anion gap metabolic acidosis and an extremely elevated osmolal gap. Toxicologic screen tests should be performed to identify the aetiology of the gap rise and proper therapy should be administered.
